# Management of convulsive status epilepticus in children: an adapted clinical practice guideline for pediatricians in Saudi Arabia

**DOI:** 10.17712/nsj.2017.2.20170093

**Published:** 2017-04

**Authors:** Fahad A. Bashiri, Muddathir H. Hamad, Yasser S. Amer, Manal M. Abouelkheir, Sarar Mohamed, Amal Y. Kentab, Mustafa A. Salih, Mohammad N. Al Nasser, Ayman A. Al-Eyadhy, Mohammed A. Al Othman, Tahani Al-Ahmadi, Shaikh M. Iqbal, Ali M. Somily, Hayfaa A. Wahabi, Khalid J. Hundallah, Ali H. Alwadei, Raidah S. Albaradie, Waleed A. Al-Twaijri, Mohammed M. Jan, Faisal Al-Otaibi, Abdulrahman M. Alnemri, Lubna A. Al-Ansary

**Affiliations:** *from the Division of Neurology (Bashiri, Hamad, Kentab, Salih, Al Nasser), Pediatric CPGs Departmental Committee (Hamad, Amer, Abouelkheir, Mohamed, Al-Ahmadi, Iqbal), Pediatric Intensive Care Unit (Al-Eyadhy), Division of General Pediatrics (Al-Ahmadi), Division of Neonatology (Alnemri), Department of Pediatrics, from the Pediatric Emergency Unit (Al Othman), Emergency Medicine Department, from the Microbiology Unit (Somily), Department of Pathology and Laboratory Medicine, from the Department of Family and Community Medicine (Al-Ansary), College of Medicine, from the Quality Management Department (Amer), Clinical Practice Guidelines Steering Committee (Amer, Abouelkheir, Iqbal, Wahabi, Al-Ansary), Research Chair for Evidence-Based Health Care and Knowledge Translation (Amer, Wahabi, Al-Ansary), Deanship of Scientific Research, from the Pediatric Clinical Pharmacy Services (Abouelkheir), King Khalid University Hospital, King Saud University Medical City, King Saud University, from the Saudi Pediatric Neurology Society (Bashiri, Kentab, Salih, Hundallah, Alwadei, Albaradie, Al-Twaijri, Jan), from the Genetic/Metabolic Division (Mohamed), Pediatric Neurology (Hundallah), Department of Pediatrics, Prince Sultan Military Medical City, from the Pediatric Neurology Department (Alwadei), National Neuroscience Institute, King Fahad Medical City, from King Abdullah Specialist Children Hospital (Al-Twaijri), National Guard King Saud bin Abdulaziz University, from the Department of Neuroscience (Al-Otaibi), King Faisal Specialist Hospital and Research Centre, from the Gulf League Against Epilepsy (Al-Otaibi), from the Saudi Epilepsy Society (Alwadei, Albaradie, Jan) Riyadh, from the Department of Pediatric Neurology (Albaradie), King Fahad Specialist Hospital, Dammam, from the Department of Pediatrics (Jan), Faculty of Medicine, King Abdulaziz University, Jeddah, Kingdom of Saudi Arabia, from the Section of Respirology (Iqbal), Department of Pediatrics and Child Health, University of Manitoba, Winnipeg, Manitoba, Canada*

## Abstract

**Objective:**

To increase the use of evidence-based approaches in the diagnosis, investigations and treatment of Convulsive Status Epilepticus (CSE) in children in relevant care settings.

**Method:**

A Clinical Practice Guideline (CPG) adaptation group was formulated at a university hospital in Riyadh. The group utilized 2 CPG validated tools including the ADAPTE method and the AGREE II instrument.

**Results:**

The group adapted 3 main categories of recommendations from one Source CPG. The recommendations cover; (i)first-line treatment of CSE in the community; (ii)treatment of CSE in the hospital; and (iii)refractory CSE. Implementation tools were built to enhance knowledge translation of these recommendations including a clinical algorithm, audit criteria, and a computerized provider order entry.

**Conclusion:**

A clinical practice guideline for the Saudi healthcare context was formulated using a guideline adaptation process to support relevant clinicians managing CSE in children.

Status epilepticus (SE) is a common and serious neurological problem in children, particularly those under 2 years of age. It is a life-threatening condition needing early recognition and intervention to prevent significant neurological sequelae. Formerly, SE was defined as a seizure lasting longer than 30 minutes or 2 or more seizures within 30 minutes, without a return to the baseline consciousness level between seizures. Recently, the definition has evolved to be convulsive seizure activity longer than 5 minutes or two or more seizures, without a return to consciousness between seizures.^1^,^2^ The incidence of SE globally is 10-58 patients per year per 100,000 population; the frequency is estimated to be 50 per year per 100,000 population, and the mortality rate to be 2.7-22%.^3^-^5^ Although there are no data on the prevalence of SE in the Kingdom of Saudi Arabia (KSA), SE in children has been identified as a high priority health topic at the King Saud University Medical City (KSUMC) based on hospital records.

Successful provision of evidence-based healthcare for patients requires accessible and trustworthy evidence-based clinical practice guidelines (CPGs) at the points of care.^6^ Currently, there are no published CPGs for SE in Saudi Arabia. Therefore, the current CPG has been suggested to be an initial, national CPG for the management of convulsive SE (CSE) in children. Members of the Saudi Pediatric Society, Saudi Pediatric Neurology Society, Saudi Epilepsy Society and the Gulf League Against Epilepsy formed a committee to review and endorse this CPG. This guideline is expected to improve the quality and safety of care for children with CSE in Saudi Arabia.

Extensive research has been conducted over the last 30 years for improving methodologies that support the synthesis of quality and trustworthy CPGs.^7^ The ADAPTE methodology for trans-contextual adaptation of CPGs has emerged as a structured, scientific, rigorous, and practical alternative to de novo development.^8^-^10^ The ADAPTE collaboration merged into the Guidelines International Network in 2009 to establish its adaptation working group, and made this version of the ADAPTE freely available on its website.^11^,^12^ This CPG adaptation project was a part of ‘The CPG adaptation program’ initiative at King Saud University. It was a collaborative endeavor between the Department of Pediatrics CPG committee and Quality Team, CPG Steering committee, Quality Management Department, and Research Chair for Evidence-Based Health Care and Knowledge Translation (CEBHC-KT).^13^-^15^

## Methods

We present a case illustration of the CPG adaptation process for management of CSE in Saudi Arabia. The Guideline adaptation group (GAG) utilized 2 CPG validated tools: the ADAPTE methodology (version 2.0), with some modifications and the AGREE II instrument.^9^,^12^,^16^ The ADAPTE consists of 3 phases.

Phase 1 (Set Up) included the selection of a high priority health topic and the formation of the GAG team. Phase 2 (Adaptation) consisted of defining clinical questions (PIPOH), identifying inclusion /exclusion criteria to search and screen source CPGs, appraising retrieved CPGs by the Appraisal of Guidelines for Research and Evaluation Instrument II (AGREE-II), and selecting from the appraised CPGs to be drafted into the finalized, adapted CPG. The AGREE-II is a validated assessment tool for the quality of CPGs, The AGREE-II includes 6 domains, namely (i) scope and purpose, (ii) stakeholder involvement, (iii) rigor of development, (iv) clarity and presentation, (v) applicability, and (vi) editorial independence.^16^ Phase 3 (Finalization) included external review of the clinical content and methodology of the adapted CPG, planning for future reviews and updates, and final production of the adapted CPG with implementation tools.

The GAG also used 3 modified and 3 new tools from the ‘Adapted ADAPTE’ CPG methods. This project was a collaborative work between clinical experts and CPG methodologists.^9^,^17^

## Results

This work marks the first CPG adaptation project for the management of CSE in children using the modified ADAPTE methodology in Saudi Arabia, an initiative from the Pediatric Neurology Unit, Department of Pediatrics at King Saud University, College of Medicine, and KSUMC.^9^,^12^

### Set up phase

The GAG was formulated in January 2013 to include three consultants, one senior registrar of pediatrics and pediatric neurology, one CPG methodologist-general pediatrician, one clinical pharmacist, supportive staff from the Department of Pediatrics’ CPG committee, a Quality Team, and the KSUMC CPG Steering Committee (Step #2). CSE was selected as a high priority topic for CPG adaptation due to the variation of practice and lack of national evidence-based CPGs. Furthermore, children with seizures were identified as among the most common categories of pediatric patient admissions in the medical city (Step #3). The GAG was aware of the relevant published CPGs and decided that the CPG adaptation was feasible (Step #1). The overall adaptation-working plan was agreed upon by the GAG (Step #6).

### Adaptation phase

The following Health questions were documented using the PIPOH model (Step #7): patient population (P); children, between 1 month and 14 years of age, with prolonged seizures; intervention (I); diagnosis and treatment; and professionals (P) such as physicians, nurses, and clinical pharmacists in the departments/specialties of pediatrics, pediatric emergency, pediatric intensive care unit (PICU), pharmacy, laboratory, and nursing. Major outcomes considered (O) were seizure control, and decreased morbidity and mortality. Healthcare setting (H) included primary, secondary, and tertiary settings in all sectors (especially pediatric inpatient wards, PICU, and emergency services).

Four main databases were searched: National Guideline Clearinghouse (NGC), Guidelines International Network (G-I-N), EBSCO DynaMed, and PubMed. Keywords used for the search were SE in children, Childhood Convulsive Status Epilepticus, and CSE (Step #8). Fifteen source CPGs were initially retrieved, and then screened using the identified PIPOH and additional inclusion/exclusion selection criteria.

The selection criteria included (i) CPGs that were evidence-based, with a detailed development methodology section or document, (ii) CPGs written in the English language, (iii) original Source CPGs, not adapted ones, (iv) CPGs that had group (organizational) rather than single authorship, and (v) CPGs that were published nationally and/or internationally between 2009 and 2013 (Steps #9 and #10). Twelve source CPGs were excluded, and three were retained for further appraisal by 2 independent appraisers (YSA, SM) using the AGREE-II instrument (Step #11) (**Table 1**). The three source CPGs selected were the Neurocritical Care Society guideline on evaluation and management of SE (NCS-2012),^18^ Texas Children’s Hospital Evidence-Based Outcomes Center: Initial Management of Seizures SE Clinical Guideline (Texas-2009) that was made available through the ‘Section on Emergency Medicine, American Academy of Pediatrics’,^19^ and the National Institute for Health and Care Excellence guideline on diagnosis and management of epilepsies in adults and children in primary and secondary care (NICE-2012).^20^,^21^ The contents of the CPGs were also compared using the free-online-tool ‘Compare Guidelines’ from the NGC.

**Table 1:** Critical appraisal of the Source CPGs by the Appraisal of Guidelines for Research & Evaluation II (AGREE II) Instrument.

CPGs AGREE-II domains scores (%)	Texas 2009	NICE 2012	NCS 2012	AES 2016
Domain 1: Scope & Purpose	92	100	78	100
Domain 2: Stakeholder Involvement	47	100	41	44
Domain 3: Rigour of Development	56	98	68	69
Domain 4: Clarity & Presentation	100	97	80	92
Domain 5: Applicability	48	85	10	21
Domain 6: Editorial Independence	50	88	19	38
Overall Assessment (%)	85	100	57	75
Recommendation for CPG use	Yes - 1, Yes with modifications - 1, No - 0	Yes - 1, Yes with modifications - 1, No - 0	Yes - 1, Yes with modifications - 0, No - 1	Yes - 1, Yes with modifications - 1, No - 0

The overall assessment and decision of the CPG Adaptation working group, based on the AGREE-II domain scores, was to adapt the NICE CPG and exclude the Texas, NCS, and AES CPGs. CPGs: Clinical Practice Guidelines, AGREE: Appraisal of Guidelines for Research and Evaluation, Texas: Texas Children’s Hospital Evidence-Based Outcomes Center, NICE: National Institute for Health and Care Excellence, NCS: Neurocritical Care Society, AES: American Epilepsy Society

**Figure 1:**
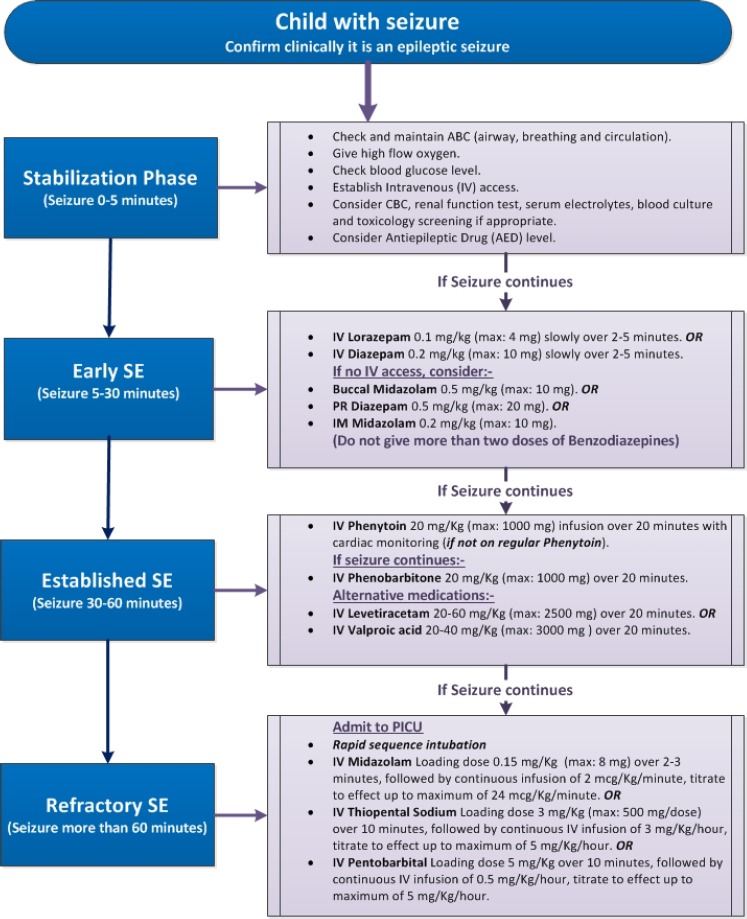
- Clinical algorithm for treatment of CSE in children.

The GAG decided to exclude NCS-2012 and Texas-2009, and include the NICE-2012 for adoption of all the recommendations related to; (i) first-line treatment of children with prolonged or repeated generalized and CSE in the community; (ii) treatment of children with CSE in the hospital; and (iii) refractory CSE. This decision was based on the compliance of the selected NICE-CPG to the AGREE-II criteria especially domain #3 (rigor of development=98%). Furthermore, the assessment of currency at the time of this study revealed that the NICE-CPG had the most updated evidence-base (the latest evidence update for the NICE-CPG was February 2014 and the latest update for the web-based NICE-CG137 guidance, as a whole, was February 2016) (Steps #12, #16-#18) (**Table 2, Figure 1**).^22^

**Table 2:** Key Recommendations of the adapted CPG for management of Convulsive Status Epilepticus (CSE) in children.

**Prolonged or Repeated Seizures and CSE**
**1.***First-Line Treatment for Children with prolonged or Repeated generalized, convulsive (Tonic–Clonic, Tonic or Clonic) Seizures in the Community*
1.1. Give immediate emergency care and treatment to children who have prolonged (lasting 5 minutes or more) or repeated (three or more in an hour) convulsive seizures in the community. 1.2. Only prescribe buccal midazolam or rectal diazepam for use in the community for children who have had a previous episode of prolonged or serial convulsive seizures. 1.3. Administer buccal midazolam as first-line treatment in children and young people with prolonged or repeated seizures in the community. Administer rectal diazepam if preferred or if buccal midazolam is not available 1.3. Treatment should be administered by trained clinical personnel or, if specified by an individually agreed protocol drawn up with the specialist, by family members or carers with appropriate training. 1.4. Care must be taken to secure the child airway and assess his or her respiratory and cardiac function. 1.5. Depending on response to treatment, the person’s situation and any personalized care plan, call an ambulance, particularly if:- 1.5.1. The seizure is continuing 5 minutes after the emergency medication has been administered. 1.5.2. The person has a history of frequent episodes of serial seizures or has CSE, or this is the first episode requiring emergency treatment or 1.5.3. There are concerns or difficulties monitoring the person’s airway, breathing, circulation or other vital signs.
**2.*Treatment for Children with CSE in Hospital***
2.1 For children and young people with ongoing generalized tonic–clonic seizures (CSE) who are in the hospital, immediately: •Secure airway •Give high-concentration oxygen •Assess cardiac and respiratory function •Check blood glucose levels and •Secure intravenous access in a large vein 2.2 Administer intravenous lorazepam as first-line treatment in children, with ongoing generalized tonic–clonic seizures (CSE). Administer intravenous diazepam if intravenous lorazepam is unavailable, or buccal midazolam if unable to secure immediate intravenous access. Administer a maximum of two doses of the first-line treatment (including pre-hospital treatment). 2.3 If seizures continue, administer intravenous phenobarbital or phenytoin as second-line treatment in hospital in children with ongoing generalized tonic–clonic seizures (CSE).
**3.*Refractory CSE***
3.1. Administer intravenous midazolam or thiopental sodium to treat children with refractory CSE. Adequate monitoring, including blood levels of Antiepileptic Drugs (AEDs), and critical life systems support are required. 3.2. As the treatment pathway progresses, the expertise of an anesthetist/intensivist should be sought. 3.3. Regular AEDs should be continued at optimal doses and the reasons for status epilepticus should be investigated. 3.4. An individual treatment pathway should be formulated for children who have recurrent CSE.
The recent SE definition included a seizure longer than 5 minutes or two or more seizures without a return of consciousness between seizures. Serial seizures are defined as 3 or more tonic-clonic seizures in an hour. SE can be divided into a number of subtypes, either by seizure type or response to treatment. Clinical SE can be either focal or generalized, and each of these types can be divided by duration into; (i) Early SE (5-30 minutes), (ii) Established SE (30-60 minutes), and (iii) Refractory SE (seizures persist despite treatment with adequate doses of two or three initial anticonvulsant medications)

### Finalization Phase

The adapted CPG was finalized and peer reviewed for clinical content by all relevant stakeholders, including 4 consultants of pediatric neurology, 2 consultants of pediatric emergency medicine, one clinical pharmacist, one consultant of pediatric critical care, and one consultant of laboratory medicine/microbiology. The adaptation process methodology was reviewed by 5 members of the CPGs Steering Committee. After the external review step (Step #19), a final version of the adapted CPG was produced and approved in April 2015. A plan for the update was agreed upon by the GAG (Steps #19-#24).

Multiple implementation tools were designed from Source CPGs and other resources, including NICE. The 4 new implementation tools included (i) a clinical algorithm (**Figure 1**), (ii) a medication table (**Table 3**), (iii) an audit criteria data collection sheet (**Table 4**), and (iv) incorporation of the hospital information system (HIS) and electronic medical records (EMR) system as a computerized provider order entry (CPOE), with order set. The patient educational material was planned to be translated into Arabic in collaboration with the Health Education Center.

**Table 3:** Drugs used for management of Convulsive Status Epilepticus (CSE) in Children.

Drug	Dose/administration	Side effects
Lorazepam	IV: 0.1 mg/kg/dose (max: 4 mg/dose) slowly over 2-5 minutes; may repeat once in 5-15 minutes if needed.	Hypotension Respiratory depression
Diazepam	IV/IO: 0.15-0.2 mg/kg/dose (max: 10 mg/dose) slowly over 2-5 minutes; may repeat once in 5-15 minutes if needed. PR: 0.5 mg/kg/dose (max: 20 mg/dose)	Hypotension Respiratory depression
Midazolam	Buccal: 0.5 mg/kg/dose (max: 10 mg/ dose) IM: 0.2 mg/kg/dose (max: 10 mg/dose) IV Infusion: loading dose: 0.15 mg/kg (max: 8 mg/dose) over 2-3 min, followed by continuous IV infusion of 2 mcg/kg/min, titrate to effect by 2 mcg/kg/min every 10-15 min up to maximum rate of 24 mcg/kg/min.	Hypotension Respiratory depression
Phenytoin	IV/IO: 20 mg/kg/dose (max: 1000 mg/dose) Dilute in normal saline. Infuse over 20 minutes. Administered into a large vein, using an in-line 0.22 micron filter. Flush IV catheter with normal saline to avoid local venous irritation and extravasation.	Hypotension Bradycardia Arrhythmias *Use with caution in cardiac patient
Phenobarbitone	IV: 20 mg/kg/dose (max: 1000 mg/dose) Infusion over 20 minutes; may repeat in 10-15 minutes if needed up to max. total dose of 40 mg/kg	Hypotension Respiratory depression
Levetiracetam	IV: 20-60 mg/kg (max: 2500 mg/dose) Infusion over 15-20 minutes	Aggression
Sodium valproate	IV: 20-40 mg/kg (max: 3000 mg/dose) infusion over 20 minutes; an additional 20 mg/kg after 10-15 min can be used if needed, followed by a continuous IV infusion of 5 mg/kg/hour. Once patient is seizure free for 6-12 hours, reduce infusion rate by 1 mg/kg/hour every 2 hours	Hyperammonemia Pancreatitis Thrombocytopenia Hepatotoxicity
Thiopental	IV: 3-5 mg/kg (max: 500 mg/dose) over 10 minutes, repeat dose if needed after 10-15 min (up to max. total dose of 10 mg/kg); followed by continuous IV infusion of 3-5 mg/kg/hour, titrate to effect.	Hypotension Respiratory depression Arrhythmias
Pentobarbital	IV: 5 mg/kg over 10 minutes, may repeat the dose if needed; followed by continuous IV infusion of 0.5-5 mg/kg/hour, titrate to effect.	Hypotension Respiratory depression Arrhythmias Paralytic ileus

CSE - Convulsive Status Epilepticus, IV - intravenous, IO - Intraosseous, IM - intramuscular, PR - Per rectal

**Table 4:** Audit data collection tool to monitor implementation of the CPG for management of CSE in children.

Items done and documented in the patient medical records			
*Diagnosis*			
Date of birth (DOB):	- - / - - / - - - - (day/month/year)		
Type of patient	◻New onset seizures ◻Known patient with seizures		
Type of seizures	◻Generalized	◻Focal	
Anti-epileptic drug (AED) levels done	◻YES	◻NO	◻Not Indicated
Toxicology screening done	◻YES	◻NO	◻Not Indicated
Blood cultures done	◻YES	◻NO	◻Not Indicated
Lumbar puncture (LP) done	◻YES	◻NO	◻Not Indicated
Electroencephalography (EEG) done	◻YES	◻NO	◻Not Indicated
*Neuroimaging studies done:*			
CT	◻YES	◻NO	◻Not Indicated
MRI	◻YES	◻NO	◻Not Indicated
*Treatment*			
Response to First-line therapy (Benzodiazepines):	◻YES	◻NO	
Response to Second-line therapy (Phenytoin):	◻YES	◻NO	
Response to Third-line therapy (Phenobarbitone):	◻YES	◻NO	
Response to Alternative therapy:	◻YES	◻NO	
*Type of medication*			
Levetiracetam	◻YES	◻NO	
Valproic acid	◻YES	◻NO	
*Response to Fourth-line therapy:-*			
Midazolam Infusion	◻YES	◻NO	
Thiopental Infusion	◻YES	◻NO	
Pentobarbital Infusion	◻YES	◻NO	
Admission to PICU	◻YES	◻NO	
Intubation/ Ventilation needed	◻YES	◻NO	
Length of stay (LOS) (Days/Hours):- •Department of Emergency Medicine (DEM) • Pediatric Intensive Care Unit (PICU) • Inpatient ward • Total LOS	--/-- --/-- --/-- --/--		
Patient outcome	◻Discharged	◻Died	
Follow up appointment in the Pediatric Neurology Clinic provided:	◻YES	◻NO	

Permission to use the NICE-CPGs in our CPG adaptation projects was obtained. National Institute for Health and Care Excellence (NICE) responded that there was no objection to our request, in principle, and the organization would be happy for the King Saud University Hospitals to adapt content from the NICE-CPGs for use in Saudi Arabia given certain specifications, including proper acknowledgments and citation. This CPG adaptation project was connected to other hospital-wide projects including (i) hospital accreditation, (ii) evidence-based practice education and training for physicians, nurses, and pharmacists, (iii) patient safety, and (iv) performance management. The recommendations were disseminated locally and nationally through educational sessions, oral presentations, discussions, and outreach visits in collaboration with the relevant national scientific specialized societies. As part of the dissemination phase, the adapted CPG was critically reviewed and endorsed by the Saudi Pediatric Association, Saudi Epilepsy Society, Saudi Pediatric Neurology Society, and Gulf League Against Epilepsy.

## Discussion

Although CSE is a common and life-threatening emergency condition, specific CPGs for the management of CSE are not widely available. Convulsive Status Epilepticus is defined as a convulsive seizure lasting longer than 5 minutes, or 2 or more seizures without a return of consciousness between seizures.^1^,^2^

The International League Against Epilepsy (ILAE) Task Force on the Classification of SE, recently defined SE as a condition resulting from either a failure of the seizure termination mechanisms or a failure of the initiation mechanisms, both which would lead to abnormally prolonged seizures (after time point t1). It is a condition that can have long-term consequences (after time point t2), including neuronal death and injury, and alteration of neuronal networks, depending on the type and duration of the seizure. This definition identified two main time points (t1 and t2). The first time point (t1) is the length of the seizure (t1); it represents ‘continuous seizure activity’ and identifies the time when treatment should be started. The second time point (t2) is the time of ongoing seizure activity beyond which there is a risk of long-term sequelae or consequences. Identification of t2 highlights the importance of SE prevention. In CSE, the identification of both time points (5 and 30 minutes for t1 and t2, respectively) was based on incomplete evidence. The ILAE also described SE to be “not a disease entity but rather a symptom with a myriad of etiologies”.^23^

The quality assessment of the 3 selected source CPGs revealed overall superior scores in the AGREE-II domains for the NICE-CPG, and the reviewers recommended to use it, with some modifications, to support implementability and provide relevant local context. These modifications included the four new implementation tools that were based on the NICE-CPG recommendations.

The CPG Steering Committee proposed cut-off points for the AGREE domain scores to facilitate the overall assessment, discussion, and decision process for the GAG. They identified weak scores (<40%), moderate scores (≥41-70%), and strong scores (≥71%). The scope and purpose (domain #1) received strong scores in all CPGs, with the NICE-CPG scoring the highest (100%). The highest quality of multidisciplinary stakeholder involvement (domain #2) was found in the NICE-CPG (100%), with moderate scores observed in the other CPGs. The highest score for rigour of development (domain #3) was obtained by NICE (98%), as it relied on a strong development methodology that is documented in detail in the NICE-CPG manual.^33^ The other CPGs scored moderately on this measure. All Source CPGs obtained strong scores for clarity and presentation (domain #4), with the Texas-CPG receiving the highest score (100%). Applicability (domain #5) scored the highest in the NICE-CPG (85%), as NICE contained 14 different categories of CPG implementation tools, including NICE pathway, quality standards, baseline assessment, clinical audit, slide set, eLearning modules, commissioning guide, tailored education support, shared learning, costing statement, case scenario, ‘do not do’ recommendations, guidance into practice, and research recommendations. The GAG decided to retain some implementation tools, as well as develop new ones, for the adapted CPG. The Texas-CPG scored moderately in applicability (48%), whereas both NCS-CPG and AES-CPG scored as weak (<40%). Editorial independence (domain #6) scored the highest in the NICE-CPG (88%) and moderate in the Texas-CPG (50%). At the time of the writing of this publication, a CPG was published by the American Epilepsy Society (AES-2016) and has received great attention from both adult and pediatric neurologists.^22^ We decided to assess its quality and to add it to the set of the three source AGREE II-assessed CPGs. It was appraised by 2 independent appraisers (YA, MH) (**Table 1**) (Step #11). The review and discussion of the AGREE II domain scores for this fourth Source CPG did not result in changing the final decision of the GAG since the NICE-CPG remained superior in most domains, particularly the rigour of development (methodology) and applicability domains. The AES did not propose any significant changes to the NICE-CPG’s recommendations.^22^ The lifecycle of this CPG adaptation project from launch to official approval was 27 months. Challenges were faced by the GAG during the adaptation process. Lessons learned from our experience may be useful to other healthcare organizations that are interested in using the ADAPTE methodology for adaptation of CPGs for CSE.

From the initial launching of this project, this CPG project was supported and promoted by the Head of the Pediatric Neurology Unit, Chairman of the Department of Pediatrics, Head of the Department Quality team, Chairperson of the CPG Departmental Committee, Chairperson of the Medical City-Wide CPG Steering Committee, Director and staff of the Quality Management Department, Director of the Hospital IT Department, and CEBHC-KT. This highlights the importance and impact of leadership commitment in CPG adaptation projects. The KSUMC organizational support throughout this CPG project was in the form of methodological, logistic, and technical consultancy rather than direct financial support. This project was not supported or funded by any industrial or pharmaceutical company.

There was an overall limited protected time for the clinical experts and methodologists involved in either the adaptation or the review of this CPG. This contributed to the prolonged duration of the project. We recommend optimizing the protected time and commissioning the contributors of the CPG adaptation projects to encourage completion of the tasks in a timely manner.

The Source CPG from NICE was originally prepared for the National Health Service in England and Wales, rather than Saudi Arabia. Nevertheless, the multidisciplinary collaborative groups for this CPG adaptation project have not identified nor anticipated any major expected barriers for implementation of the NICE recommendations, mainly including pharmacological therapy using Anti-epileptic drugs (AEDs) in the Saudi Arabian healthcare setting. Furthermore, the KSA is classified, like the UK, as a ‘high-income country’, according to the updated World Bank list of economies. This similarity in economic health reflection was taken into consideration during the trans-contextual adaptation process.^24^

This type of CPG adaptation project has multi-faceted purposes for healthcare providers and organizations. It can be utilized simultaneously for healthcare quality improvement, a research platform, continuous professional development, and an evidence-based practice project. It forms a win-win paradigm for all stakeholders, including clinicians, healthcare providers, methodologists, policy makers, and ultimately for patients, who are at the center of care.

### Implications for practice

Apart from CPG adaptation, the AGREE-II as a standalone instrument can be used by general pediatricians and pediatric neurologists to critically appraise any CPG. The adapted CSE-CPG can support performance improvement, help general pediatricians and pediatric neurologists to make informed, evidence-based clinical decisions, decrease variation in practice, and avoid non-indicated investigations or treatments for infants and children with CSE. The 4 implementation tools reported can be adopted and used by any child healthcare provider and/or facility in KSA except for the in-house developed CPOE with order set. The CPOE will need to be rebuilt and/or customized according to the functionalities of the local HIS/EMR.

### Management of CSE in children. Prehospital management

Early treatment for CSE is associated with cessation of the convulsion and an improved outcome; treatment should start prehospital, at home or in the community, with the administration of buccal midazolam or rectal diazepam. Prehospital management is associated with a shorter SE duration.^25^ Buccal midazolam is as effective as rectal diazepam in terminating the acute attack of the seizure.^26^,^27^ Discussing administration of these medications with the parents of any child with epilepsy is crucial. Prehospital medications, should be made available for use in the ambulance and emergency care services, and be prescribed for children who have a history of frequent seizures. Paramedical personnel should also be trained to administer these medications.

### In hospital management

SE is a life-threatening neurological emergency requiring urgent intervention. The successful management of CSE in hospital depends on the rapid use of adequate doses of AEDs.^28^ Failure of early intervention has been associated with increased need for intensive care admission and serious complications.^29^

All efforts should be directed towards stabilization of the patient and termination of the seizure. These include support of the airway, breathing, and circulation, providing proper positioning of the patient and high flow oxygen, and measuring blood glucose. Intravenous (IV) access should be established immediately. Benzodiazepines remain the drug of choice as a first line therapy, having a high strength of evidence;^18^,^20^,^22^ IV lorazepam and diazepam are effective initial medications.^30^,^31^ A recent randomized control trial did not support the preferential use of lorazepam over diazepam.^32^ Buccal or intramuscular midazolam, or rectal diazepam can be used if the IV line cannot be established rapidly. Continuous monitoring is required as the use of benzodiazepines may result in hypotension and respiratory depression. If a seizure continues 5-10 minutes from the initial dose, then a second dose can be administered.

### Second-line therapy

Evidence-based recommendations for second-line therapy for CSE do not currently exist. Phenytoin, fosphenytoin, phenobarbital, valproic acid, levetiracetam, and recently lacosamide, are frequently prescribed for the treatments of CSE.^33^ Although fosphenytoin has greater tolerability than phenytoin, the availability and cost of fosphenytoin in KSA precludes its recommendation in this CPG. The NICE-CPG recommends the use of either phenytoin or phenobarbitone as second-line therapy; the AES-CPG also recommends phenobarbitone if phenytoin, valproic acid, or levetiracetam are not available.^21^ Valproic acid is as effective as phenytoin in aborting CSE.^34^ While recognizing the side effects of valproic acid, especially in children less than two years of age due to hepatotoxicity, and a major concern in children with inborn errors of metabolism, particularly those with mitochondrial disorder,^34^ valproic acid is still considered as one of the alternative therapies. Levetiracetam is a promising drug in aborting CSE with an efficacy of 68.5%.^35^ However, there are no head-to-head studies comparing levetiracetam to other AEDs.

### Refractory CSE

Refractory status epilepticus (RSE) occurs when a seizure fails to respond to both first and second line therapy. Treatment options include midazolam infusion, sodium thiopental, or pentobarbital.^18^,^20^,^28^ Treatment of RSE requires intubation and admission to the PICU.

### Future directions

Due to lack of evidence in the treatment of CSE, many clinical trials are ongoing. Emergency Treatment with Levetiracetam or Phenytoin in status epilepticus in Children (EcLiPSE) is an open-label randomized multicenter control trial in UK comparing the efficacy of levetiracetam and phenytoin in children who fail to respond to the first line treatment (Benzodiazepine). The Established Status Epilepticus Treatment Trial (ESETT), which compares the efficacy of fosphenytoin, levetiracetam, and valproic acid in benzodiazepine refractory status epilepticus, is currently enrolling patients.^38^-^40^ The recently published evidence-based treatment CPGs by the American Epilepsy Society addressed the importance of conducting additional multicenter and multinational, randomized controlled studies to clarify important clinical unresolved questions and improve the evidence.^22^

In conclusion, the modified ADAPTE methodology for CPG adaptation was useful and practical in promoting evidence-based healthcare, reducing duplicative efforts needed to develop a CPG de novo, and promoting the local uptake of high-quality CPG recommendations without the need for substantial time or resources. An adapted CPG for the Saudi healthcare system was formulated using a CPG adaptation process to support relevant clinicians and healthcare providers during evidence-based healthcare provision for children with convulsive status epilepticus.
